# Training Health Care Practitioners to Effectively Communicate With Older Adults With Cancer and Their Caregivers

**DOI:** 10.1200/OP-25-00546

**Published:** 2025-09-19

**Authors:** Patricia A. Parker, Smita C. Banerjee, Yesne Alici, Christian J. Nelson, Koshy Alexander, Elizabeth Schofield, Faith S. Fasakin, Nessa Coyle, Andrew J. Roth, Ruth Manna, Javier Gonzalez, Rebecca Ewert, Beatriz Korc-Grodzicki

**Affiliations:** ^1^ Department of Psychiatry & Behavioral Sciences, Memorial Sloan Kettering Cancer Center, New York, NY; ^2^ Department of Psychiatry, Weill Cornell Medical College, New York, NY; ^3^ Department of Medicine, Memorial Sloan Kettering Cancer Center, New York, NY; ^4^ Department of Medicine, Weill Cornell Medical College, New York, NY; ^5^ Patient Representatives, Memorial Sloan Kettering Cancer Center, New York, NY; ^6^ Department of Neurology, Memorial Sloan Kettering Cancer Center, New York, NY

## Abstract

**PURPOSE:**

Providing medical care to older adults with cancer is complex. In addition to their cancer diagnosis, many older adults have various factors such as frailty, comorbidities, cognitive decline, sensory and functional issues, and polypharmacy that increase the complexity of their medical care. However, few health care practitioners (HCPs) receive adequate training in geriatric principles or in effective communication with older adults with cancer and their caregivers.

**METHODS:**

To meet this significant and growing need, we developed and conducted an evidence-based educational training program in geriatric oncology, Geriatric Oncology: Cognition and Communication (Geri Onc CC). The goal of this study was to examine its efficacy in terms of participants' ratings of the program, self-efficacy, knowledge, and attitudes, as well as skill uptake of general and geriatric-specific communication skills. Participants completed questionnaires and standardized patient and caregiver assessments before and after their participation.

**RESULTS:**

Two hundred eighty-two HCPs representing more than eight disciplines, working in varied types of health care settings and locations, participated in the 2-day in-person or virtual training program. Participants rated the program as having high value and reported high satisfaction. After participating in the program, they demonstrated increased knowledge (*P* < .001), greater self-efficacy (*P* < .001), and significant increase in communication skills use from pre- to post-training in five skill categories (agenda setting, checking, questioning, information organization, and empathy) as well as geriatric-specific skills (all *P* values < .001).

**CONCLUSION:**

The Geri Onc CC Training Program improved participants' knowledge and skills. This program fills an important gap in HCPs' education for this population.

## INTRODUCTION

The number of older adults with cancer continues to increase. Among an estimated 19.3 million new cancers worldwide, 65% were among individuals age 60 years or older, and older adults accounted for more than 71% of cancer-related deaths, a number expected to grow.^1^

**CONTEXT**

**Key Objective**
We assessed the efficacy of a training program (Geriatric Oncology: Cognition and Communication; Geri Onc CC) for multidisciplinary health care practitioners (HCPs) that focused on meeting the unique needs of older adults with cancer and their caregivers.
**Knowledge Generated**
HCPs favorably rated the Geri Onc CC program. Attending the program was associated with increased knowledge, self-efficacy, and communication skills.
**Relevance**
The Geri Onc CC program is efficacious and fills an important gap in the training of HCPs caring for older adults with cancer and their caregivers.

Cancer care is complex and is especially so for older adults because of factors such as frailty, comorbidities, cognitive decline, sensory and functional issues, and polypharmacy.^2-4^ These factors significantly influence treatment decisions and cancer care.^5-9^ In response, several organizations, including ASCO, the US National Comprehensive Care Network, the International Society of Geriatric Oncology, and the European Society for Medical Oncology, have established guidelines and priorities for the care of older adults with cancer that incorporate the impact of these and related factors.^10-13^

Despite the growing prevalence of older adults with cancer, few health care practitioners (HCPs) receive any formal training in geriatric oncology—a critical barrier to high-quality care.^14-16^ Extermann et al^10^ identified education as a top priority, recommending the integration of geriatric oncology into health professional training, along with accessible continuing education (eg, virtual or e-learning) for HCPs in medicine, nursing, and allied health fields. Others have similarly advocated for expanded training across oncology disciplines.^17^ The National Academy (formerly the Institute of Medicine) report, “Delivering High Quality Cancer Care,” emphasized the urgent need to prepare the workforce for an aging population, highlighting widespread gaps in incorporating geriatric principles.^18^

In addition to geriatric principles, effective communication with older adults and their caregivers is vital.^19^ Yet, few HCPs receive specific training in communication skills, particularly those tailored to the needs of older patients, and specialized training opportunities remain limited. To address this gap, we developed a 1-day three-module geriatric communication skills training program. The three modules were Geriatrics 101,^20^ Cognitive Syndromes,^21^ and Shared Decision Making.^22^ Development of the modules and preliminary efficacy of the communication skills training program are published elsewhere.^23^ Because cancer care for older adults is complex, a training focused on communication skills alone is not ideal. Education in key content areas such as cognition and its impact on cancer care needs attention.

Therefore, we developed and implemented Geriatric Oncology: Cognition and Communication (Geri Onc CC), an evidence-based training program funded by the National Cancer Institute. The curriculum was initially developed and piloted as part of a grant awarded to the last author (B.K.-G.) from the Health Resources and Services Administration, Geriatric Workforce Enhancement Program. It included an interprofessional group with representation from medicine, psychiatry, geriatrics, occupational and physical therapy, pharmacy, nutrition, nursing, social work, immigrant health, as well as community partners. The following steps guided its development: systematic literature review, expert opinion, and consensus review. Educational programs were evaluated using course evaluations and pre- and post-test knowledge assessments. The programs were highly rated and there were significant improvements in knowledge.^24,25^ For the communication modules, we followed a development approach used in previous research: (1) systematic literature review, (2) consensus review, (3) modular blueprint development, (4) training materials development, (5) scenario development, (6) revising and adapting iteratively, and (7) assessment.^26^ The multilevel Kirkpatrick assessment mode^27^ was used for evaluation.^23^

The Geri Onc CC training program was developed for HCPs from all disciplines working with older adults with cancer, with a primary focus on cognition and on teaching and modeling effective communication strategies for this population. We have previously published on the development of the program and its content and format, including some preliminary outcomes, especially participants' satisfaction ratings.^28^ The current article further examines the efficacy of the Geri Onc CC program in terms of both HCP-reported (self-efficacy, knowledge, and attitudes) outcomes and objectively evaluated communication skills (both general and geriatric-specific communication skills).

## METHODS

### Geriatric Oncology Cognition and Communication Training Program

**FIG 1.**
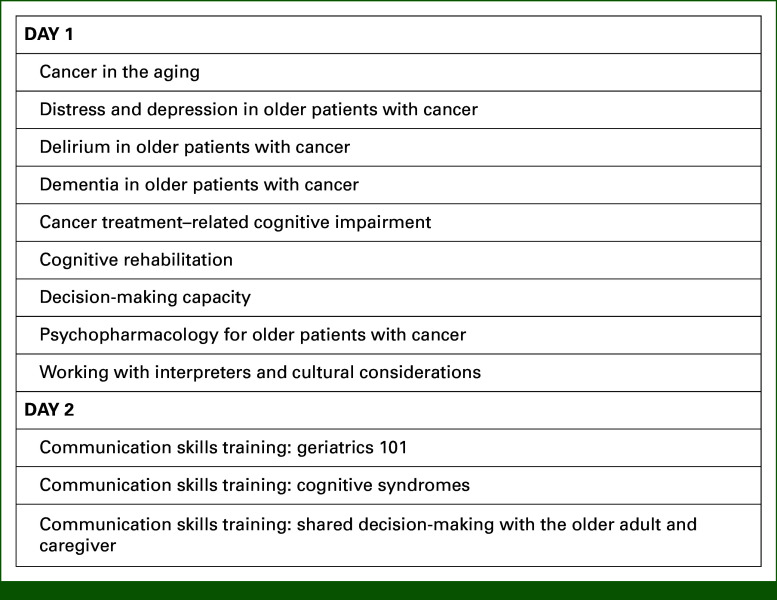
Geriatric oncology: cognition and communication curriculum.

Participants attended a 2-day virtual or in-person training followed by 12 months of ongoing consolidating learning sessions (Fig 1). Day one consisted of a series of didactic lectures, panel discussions, and video demonstrations on topics related to caring for older adults with cancer, focusing on geriatric syndromes and the influence of cancer on these with an emphasis on cognitive syndromes. The second day included three communication skills training modules in which participants put into practice what they learned on day one in simulated role play with professional, trained actors who portrayed patients and caregivers.^23^ Additional details about the development of the training and the content have been published elsewhere.^28^

### Participants and Procedures

Briefly, HCPs were recruited through e-mail to colleagues, postings on listservs, and through oncology and medicine organizations and discipline-specific organizations. We also advertised through our institution's Continuing Medical Education office and social media. Interested HCPs completed an online application about their experience, background, and interest. Participants were selected and prioritized on the basis of whether they provided direct care to older adults with cancer and geriatric syndromes, worked with underserved populations, and committed to complete all training activities. Participants also needed to speak, read, and understand English proficiently. Trainings were completed in cohorts of about 20 HCPs, and we attempted to group participants so that there was heterogeneity in discipline and clinical setting.

Before training, participants reported their demographic and work characteristics, and were assessed for knowledge, attitudes, and self-efficacy. After training, participants completed course evaluations and were again assessed for knowledge, attitudes, and self-efficacy. Participants also completed standardized patient and caregiver assessments^29^ (SPAs; ie, video-recorded interactions in which trained actors portray a patient and caregiver in a clinical scenario with the HCP to assess communication skill usage) before and after training. Participants received no compensation for participating in the training or completing the assessments.

### Measures

#### Demographic and Work Characteristics

Participants were asked to provide their race/ethnicity, sex, discipline, academic degree, and experience, as well as the setting in which they work and population they serve (ie, percent of older adults and race/ethnicity characteristics).

#### Evaluation of the Training Content

Immediately after the 2-day training, participants rated the perceived clarity, quality, and value of the training, and whether they believed the training increased their knowledge and met their goals on a five-point Likert scale (1 = strongly disagree to 5 = strongly agree). There were also 10 questions that asked participants how well the objectives in specific areas were met on a 1 (poor) to 3 (excellent) scale. These items were developed for this training since it was specific to the content of the training components. HCPs were also invited to share open-ended responses about their experience with the program.

#### Knowledge About Geriatric Oncology

Knowledge was assessed with 10 multiple choice questions written by the faculty presenters of each topic directly reflecting the course content. We reviewed items for clarity, and they were pretested on a group of 5 HCPs.

#### Attitudes

Attitudes toward older individuals in general and caring for older patients were assessed using the 14-item UCLA Geriatric Attitudes Scale.^30,31^ Scores ranged from 1 = strongly disagree to 5 = strongly agree, and a composite store was calculated. Higher scores indicated more favorable attitudes toward older patients.

#### Self-efficacy

The self-efficacy scale was developed by the authors and has been used in previous studies of communication skills training including geriatric communication skills trainings.^23,32,33^ HCPs indicated their perceived ability to complete the tasks of each module on a five-point Likert scale (1 = strongly disagree to 5 = strongly agree).

#### Communication and Geriatric Skills Uptake

Uptake of skills was assessed by evaluation of encounters of the HCPs during SPAs that were completed before and after training. This involved a 12-minute video-recorded interaction between the HCP and the trained standardized patient and caregiver using a relevant clinical scenario that provides in vivo practice in skills taught in the training. Trained coders, not part of the study and blinded to whether it was a pre- or post-SPA, used a standardized coder rating scale, the Comskil Coding System (CCS),^33^ which has been modified to include geriatric-specific communication skills.^23^ Coders were trained and overseen by one of the study authors (S.C.B.). The SPAs were analyzed using the presence or absence of 39 communication skills in six categories (agenda setting, checking, questioning, information organization, empathic communication, and geriatric-specific communication). The institution's institutional review board (IRB) determined that this research was exempt from review. Annual reports were submitted as required by the IRB, and participants were informed of all study assessments before participation.

### Data Analysis

Summary statistics were used to describe participant demographics, module evaluations, and overall satisfaction. Open-ended questions were not formally coded but were used to make improvements to the program. Changes in participants' knowledge, attitudes, and self-efficacy were assessed from pretraining to post-training using a series of mixed-effects regression models, where change scores were regressed on both an overall intercept and a cohort-specific intercept. This conservative approach allowed us to test for a significant change using the overall intercept, while adjusting for the clustered nature of outcomes within training cohorts. We hypothesized that knowledge, attitudes, and self-efficacy would increase significantly from pretraining to post-training. An effective sample size of n = 113 provides 80% power to detect a standardized effect size of at least d = 0.27 for testing a change score against a null hypothesis of no change, assuming the intraclass correlation within training cohorts is no >0.08. This effective sample size was attained with a nominal sample of 282.

Uptake of skills was assessed by evaluating the encounters of the HCPs during SPAs. All video-recorded SPAs were analyzed using an adapted version of the CCS, which codes the presence of the communication skills taught in the Geri Onc CC training program (described above).^23,33^ For analysis, items were grouped into six categories (eg, empathic communication, information organization, geriatric-specific skills, and summed, following scoring procedures from past trainings). Statistical analyses were conducted in SAS version 9.4 with alpha set to .05.

## RESULTS

Characteristics of the 282 HCP participants are shown in Table 1. Participants were 87% female. Sixty-six percent were White and 67% non-Hispanic. Participants represented more than eight disciplines with most being social workers (24%), physicians (20%), and nurse practitioners (13%). They worked in a variety of settings, including comprehensive cancer centers (41%), community hospitals/clinics (21%), and academic medical centers (17%). One third (33%) had been caring for older adults with cancer for 1-5 years, with another one quarter (24%) having done so for 6-10 years. Eighty-five percent participants indicated that more than half of their patients were older than 65 years. Ninety-two percent indicated that they at least occasionally experienced communication challenges with their patients and 43% reported that they have communication challenges with patients at least weekly. Twenty-five percent of participants had previously had some type of training related to communication with patients. All 282 participants completed the training; 99% completed the pretraining questionnaires and 98% completed the pretraining SPA. Ninety-six percent completed the post-training questionnaire and 93% the post-training SPA.

**TABLE 1.** Participant Demographic and Professional Characteristics (N = 282)

Characteristic	No. (%)
Sex^a^	
Female	244 (87)
Male	35 (12)
Transgender woman	1 (<1)
Genderqueer/nonconforming	3 (1)
Ethnicity	
Hispanic	46 (16)
Non-Hispanic	189 (67)
Something else	38 (13)
Refused	9 (3)
Race	
White	186 (66)
Asian	46 (16)
Black/African American	16 (6)
Native American	2 (1)
Other	20 (7)
Refused	12 (4)
Clinical area	
Oncology	118 (42)
Social work	36 (13)
Geriatrics	30 (11)
Psychiatry	18 (6)
Palliative care	16 (6)
Nursing	14 (5)
Physical therapy	11 (4)
Occupational therapy	7 (2)
Internal medicine	2 (1)
Nutrition	1 (0)
Other	29 (10)
>50% of their patients	
Over age 65 years	241 (85)
Hispanic/Latino	53 (19)
Asian/Pacific Islander	14 (5)
African American/Black	46 (16)
Caucasian/White	217 (77)
Native American	4 (1)
Other/unknown	16 (6)
Discipline (academic degree)	
Social worker	68 (24)
Physician	57 (20)
Nurse practitioner	36 (13)
Nurse	35 (12)
Psychologist	29 (10)
Physical therapist	10 (4)
Occupational therapist	9 (3)
Physician assistant	4 (1)
Dietician	1 (0)
Other	33 (12)
How long caring for older patients with cancer?	
Never, but interested	6 (2)
Less than 1 year	22 (8)
1-5 years	93 (33)
6-10 years	67 (24)
11-15 years	41 (15)
16+ years	53 (19)
Attended previous communication skills training?	
Yes	70 (25)
No	212 (75)
Employment setting	
Comprehensive cancer center	69 (24)
Community hospital/clinic	60 (21)
NCI-designated cancer center	52 (18)
Academic medical center	49 (17)
Freestanding cancer center	10 (4)
Mental health clinic	7 (2)
Private practice	5 (2)
Hospice	5 (2)
Veteran Affairs hospital	3 (1)
Other	22 (8)
Communication challenges with patients	
Never	1 (<1)
Rarely (a few times per year)	22 (8)
Occasionally (monthly)	137 (49)
Very often (weekly)	94 (33)
All the time (daily)	28 (10)

Abbreviation: NCI, National Cancer Institute.

^a^ Note that multiple sex categories could be selected.

After the training, participants rated all lectures and roleplay sessions (on a scale from 1 to 5, with 5 being more favorable) as having high clarity (mean [M] = 4.8, standard deviation [SD] = 0.4), quality (M = 4.8, SD = 0.4), and value (M = 4.7, SD = 0.4), and indicated that they believed that the training increased their knowledge in key areas (M = 4.7, SD = 0.5) and that the training met their goals (M = 4.70, SD = 0.4). Scores on individual sessions were generally high. Across sessions, mean clarity ratings were at least 4.6, quality at least 4.6, value at least 4.3, increased knowledge at least 4.5, and met goals at least 4.4. Means for all aspects (ie, clarity, quality) on each session are available in Appendix Table A1 (online only). Participants' overall satisfaction with the course averaged 3.8 (SD = 0.4) on a 1-4 scale, with a higher score indicting higher satisfaction. In terms of how the course met objectives in specific areas of geriatric oncology, average scores ranged from 2.7 to 2.9, on a 1-3 scale, with a higher score indicating that the course met their objectives.

Open-ended responses about the program focused on many different aspects of the program. Participants indicated the ways in which their care of patients would be improved such as “My comprehensive assessment of patients will be greatly improved with the addition of cognitive assessment as a routine for older patients.” Others focused on the changes that they anticipate when interactions with patients. Examples included, “I have known for a while that I could learn to be more efficient in my interactions with patients and their families. This provided me with a structured framework to use to direct interactions and help me stay on task better,” “I feel this program provided me with the necessary tools to effectively communicate with geriatric patients and their families in my role and I am very grateful that I attended,” and “I am definitely more confident in working to achieve goal concordant care for our patients based on the challenges they encounter as older adult patients with cancer.”

**FIG 2.**
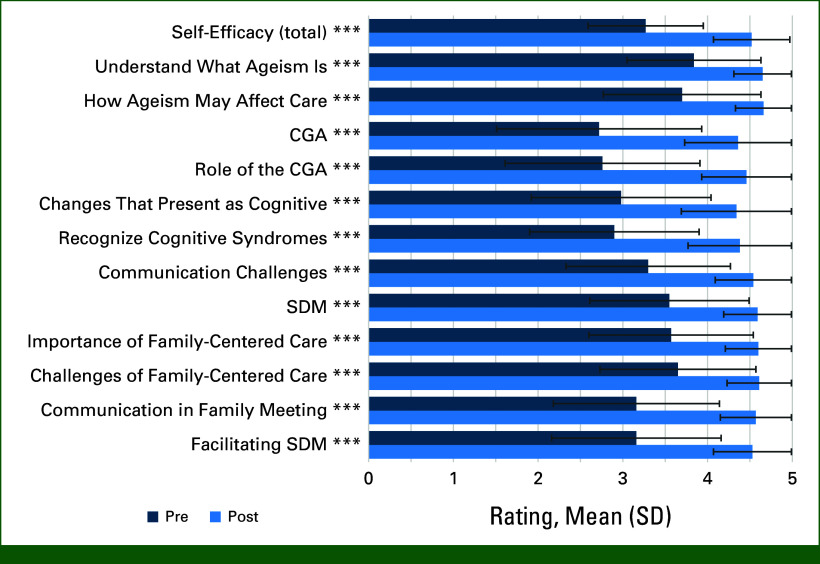
Self-efficacy total and individual items before and after training. ***Indicates the pre-post paired comparison is significant at the .001 level. CGA, Comprehensive Geriatric Assessment; SD, standard deviation; SDM, shared decision making.

Participants indicated a significant increase in self-efficacy regarding various aspects of geriatric oncology after the course compared with their precourse self-efficacy ([premean = 3.3, SD = 0.7; postmean = 4.5, SD = 0.5], *P* < .001). Distributions of individual self-efficacy items and total score are depicted in Figure 2. They also had significant increases in knowledge about aspects of geriatric oncology taught in the course ([premean = 6.1, SD = 1.6; postmean = 6.7, SD = 1.5], *P* < .001). Scores about attitudes toward older adults did not change ([premean = 4.1, SD = 0.4; postmean = 4.1, SD = 0.6], *P* > 1.0).

On the basis of blinded coding of the patient-caregiver SPAs, before and after the training, participants demonstrated a significant increase in communication skills use from pre- to post-training in total skills ([premean = 6.2, SD = 2.3; postmean = 7.9, SD = 2.6], *P* < .001), as well as in the individual skill categories: agenda setting ([premean = 0.5, SD = 0.6; postmean = 1.3, SD = 0.9], *P* < .001; possible range 0-4), checking skills ([premean = 0.6, SD = 0.7; postmean = 0.8, SD = 0.7], *P* < .001; possible range 0-2), questioning skills ([premean = 2.4, SD = 0.9; postmean = 2.8, SD = 0.9], *P* < .001; possible range 0-5), information organization ([premean = 0.4, SD = 0.7; postmean = 0.6, SD = 0.7], *P* < .001; possible range 0-4), and empathy ([premean = 2.2, SD = 1.3; postmean = 2.6, SD = 1.3], *P* < .001; possible range 0-5). A significant increase in geriatric-specific skills was also seen ([premean = 7.8, SD = 1.8; postmean = 8.7, SD = 1.6], *P* < .001; possible range 0-19). Means of individual skills are listed in Table 2.

**TABLE 2.** Skill Use in Standardized Patient and Caregiver Assessments by Pre- or Post-Training

Category or Skill	Pre (n = 276)	Post (n = 254)	*P*
Communication skills, overall [0-20], mean (SD)	6.2 (2.3)	7.9 (2.6)	<.001
Agenda setting [0-4], mean (SD)	0.5 (0.6)	1.3 (0.9)	<.001
Declare agenda, No. (%)	120 (43)	183 (72)	<.001
Invite agenda, No. (%)	17 (6)	107 (42)	<.001
Negotiate agenda, No. (%)	7 (3)	27 (11)	<.001
Take stock, No. (%)	3 (1)	5 (2)	.480
Checking skills [0-2], mean (SD)	0.6 (0.7)	0.8 (0.7)	.006
Check understanding, No. (%)	104 (38)	111 (44)	.166
Check preference, No. (%)	63 (23)	80 (31)	.004
Questioning [0-5], mean (SD)	2.4 (0.9)	2.8 (0.9)	<.001
Ask open-ended questions, No. (%)	266 (96)	248 (98)	.317
Clarify, No. (%)	177 (64)	159 (63)	.581
Restate, No. (%)	144 (52)	175 (69)	<.001
Endorse question-asking, No. (%)	22 (8)	39 (15)	.002
Invite questions, No. (%)	65 (24)	82 (32)	.021
Information organization [0-4], mean (SD)	0.4 (0.7)	0.6 (0.7)	<.001
Preview information, No. (%)	21 (8)	33 (13)	.025
Summarize information, No. (%)	18 (7)	19 (7)	.433
Transition, No. (%)	29 (11)	41 (16)	.083
Review next steps, No. (%)	36 (13)	56 (22)	.005
Empathic communication [0-5], mean (SD)	2.2 (1.3)	2.6 (1.3)	<.001
Encourage expression of feelings, No. (%)	159 (58)	155 (61)	.345
Acknowledge, No. (%)	147 (53)	172 (68)	<.001
Validate, No. (%)	172 (62)	172 (68)	.292
Normalize, No. (%)	53 (19)	70 (28)	.013
Praise efforts, No. (%)	74 (27)	81 (32)	.204
Geriatric oncology communication skills [0-19], mean (SD)	7.8 (1.8)	8.7 (1.6)	<.001
Clinician introduced themselves, No. (%)	242 (88)	218 (86)	.564
Greeted patient and caregiver, No. (%)	210 (77)	236 (93)	<.001
Talked to patient, not about them, No. (%)	259 (95)	252 (100)	<.001
Took patient permission, No. (%)	68 (25)	102 (40)	<.001
Mentioned the GA, No. (%)	3 (1)	5 (2)	.317
Described the GA, No. (%)	11 (4)	13 (5)	.467
Asked about ADLs, No. (%)	164 (60)	164 (65)	.136
Asked about IADLs, No. (%)	119 (43)	157 (62)	<.001
Introduced cognitive assessment, No. (%)	54 (20)	50 (20)	.819
Conducted cognitive assessment, No. (%)	34 (12)	34 (13)	.586
Made partnership statements, No. (%)	153 (56)	163 (64)	.008
Elicited patient perspective, No. (%)	270 (99)	251 (99)	.414
Elicited caregiver perspective, No. (%)	243 (89)	244 (96)	.001
Presented treatment recommendation, No. (%)	67 (24)	58 (23)	.833
Suggested referrals, No. (%)	194 (71)	191 (75)	.335
Judgmental tone—memory/cognition, No. (%)	13 (5)	6 (2)	.225
Offered decision delay, No. (%)	34 (12)	44 (17)	.204
Dismissal—ageist remark, No. (%)	1 (0)	5 (2)	.103
Use of elderspeak	10 (4)	4 (2)	.132

NOTE. Item *P* values are based on McNemar's test for paired dichotomous data; thus, pretraining SPAs are excluded if no matching post-training SPA available. Category *P* values are based on paired-samples *t*-tests.

Abbreviations: ADL, activities of daily living; GA, geriatric assessment; IADL, instrumental activities of daily living; SD, standard deviation; SPA, standardized patient and caregiver assessments.

## DISCUSSION

The Geri Onc CC program was found to be highly valued by HCPs of various disciplines. Nearly all (92%) participants indicated that they had at least occasionally and 43% weekly experienced communication challenges with older patients with cancer before the training, yet only 25% had previously had any kind of communication skills training. Efficacy of the program was demonstrated in many areas including knowledge and self-efficacy. Importantly, in addition to the self-report measures, significant changes were also found in the use of communication skills in simulated encounters with patients and caregivers as rated by blinded coders.

There were no changes in attitudes toward older adults. HCPs' attitudes were already highly positive before participating in the program. This is not surprising, given that these participants sought out additional training in working with older adults. According to the developers of the measure, a mean score higher than three indicates positive attitudes toward older adults.^31^

We believe many aspects of our training program led to its success. We recruited HCPs from various disciplines. The field of geriatric oncology is multidisciplinary, and our program replicates this structure and the recommendations and guidelines for optimal care of older patients with cancer.^34^ Participants frequently commented on the benefits of learning with HCPs from other disciplines. This not only creates better understanding of the other areas, but it may also improve the overall communication and information provided to older patients with cancer as HCPs from different disciplines are learning similar content and communication skills. Participants varied in many other ways. There was heterogeneity in race/ethnicity of participants; they practiced in 38 states and Washington, DC in the United States and from 18 countries, worked in different settings, and many provided care to many patients from underrepresented backgrounds. The training is interactive and allows participants to practice the content and skills they are learning (with standardized patient and caregiver actors) so that they feel more prepared and confident in their own settings.

This program fills an essential gap in older adult care. We received many more applications for the program than we could accommodate (eg, 428 HCPs applied in a 16-month period when we could only admit 110). This highlights the great need to improve knowledge and communication through this program or other similar educational opportunities. The HCPs who participated were actively engaged throughout and discussed their plans to share the knowledge with others at their workplaces. Future work could investigate interprofessional communication about the training program after participation and whether it reduces stress and burnout among HCPs.

Some limitations should be noted about the study. First, we did not conduct a needs assessment of the target population to understand their individual challenges. Given our decade-long work in this area, we included the content areas that were significant from our previous work and the literature. Second, we did not include an assessment of the HCP with patients in their clinic setting. Given the nature of the program and that the participants came from many different institutions, states, and countries, this was not feasible. One advantage, however, of using SPAs is that we can ensure that there is an opportunity for HCPs to use communication skills that were taught and that we can ensure that a caregiver is present during the encounter. The semistructured nature of SPAs permits more control over the content of the encounter and appraisal of all desired skill elements of the Geri Onc CC training compared with clinic visits, which could be highly variable.

We were unable to determine whether positive changes in HCPs' communication skills were maintained over time. We received additional funding to continue the program, and future learners will complete an additional SPA 6 months after they participate in the 2-day program. This will allow us to evaluate whether these improvements persist over time. Future work will also examine whether other improvements seen after the training are maintained and how they influence care for older patients and caregivers.

The demand for this program clearly demonstrates the tremendous need and eagerness among oncology HCPs for training opportunities in geriatric oncology. Learning more about the unique needs of older adult patients with cancer, including improving communication skills, will allow for better patient-centered care for this growing population.

## APPENDIX

**TABLE A1.** Participants' Ratings of Clarity, Quality, Value, Increase in Knowledge, and Attainment of Goals for Individual Sessions and Overall (n = 273)

Session	Clarity, Mean (SD)	Quality, Mean (SD)	Value, Mean (SD)	Increased Knowledge, Mean (SD)	Met Goals, Mean (SD)
Cancer in the aging	4.8 (0.4)	4.9 (0.4)	4.8 (0.5)	4.7 (0.5)	4.8 (0.5)
Identifying and measuring depression	4.8 (0.5)	4.8 (0.5)	4.8 (0.4)	4.8 (0.5)	4.8 (0.5)
Delirium in older patients with cancer	4.8 (0.4)	4.8 (0.4)	4.8 (0.5)	4.7 (0.5)	4.8 (0.4)
Treatment related cognitive impairment	4.8 (0.5)	4.8 (0.5)	4.8 (0.4)	4.8 (0.5)	4.8 (0.5)
Cognitive rehabilitation	4.7 (0.6)	4.7 (0.7)	4.7 (0.7)	4.6 (0.7)	4.7 (0.7)
Decision-making capacity	4.8 (0.5)	4.8 (0.5)	4.8 (0.5)	4.8 (0.5)	4.8 (0.5)
Psychopharmacology	4.7 (0.6)	4.7 (0.6)	4.6 (0.8)	4.6 (0.7)	4.6 (0.7)
Addressing the language barrier	4.7 (0.5)	4.7 (0.5)	4.7 (0.6)	4.5 (0.7)	4.6 (0.7)
Responsible conduct of research	4.6 (0.6)	4.6 (0.7)	4.3 (0.9)	NA	4.4 (0.9)
Comskil conceptual model	4.8 (0.5)	4.8 (0.5)	4.8 (0.5)	NA	4.8 (0.5)
Geriatrics 101	4.8 (0.5)	4.8 (0.5)	4.9 (0.5)	NA	4.8 (0.5)
Geriatrics 101 roleplay	4.8 (0.5)	4.8 (0.5)	4.8 (0.5)	NA	4.8 (0.5)
Cognitive syndromes	4.8 (0.5)	4.8 (0.5)	4.8 (0.5)	NA	4.8 (0.5)
Cognitive syndromes roleplay	4.8 (0.6)	4.8 (0.5)	4.8 (0.6)	NA	4.8 (0.6)
Shared decision making	4.8 (0.5)	4.8 (0.5)	4.8 (0.5)	NA	4.8 (0.5)
Shared decision-making roleplay	4.8 (0.5)	4.8 (0.5)	4.8 (0.5)	NA	4.8 (0.5)
Group think tank	4.6 (0.7)	4.6 (0.7)	4.6 (0.7)	NA	4.5 (0.8)
What happens next	4.7 (0.6)	4.7 (0.6)	4.7 (0.7)	NA	4.6 (0.7)
Mean across all sessions	4.8 (0.4)	4.8 (0.4)	4.7 (0.4)	4.7 (0.5)	4.7 (0.4)

NOTE. Each rating has a possible range of 1 to 5, with 5 being most favorable.

Abbreviation: NA, not applicable.
